# Cuproptosis in glioblastoma: unveiling a novel prognostic model and therapeutic potential

**DOI:** 10.3389/fonc.2024.1359778

**Published:** 2024-03-28

**Authors:** Zhigang Qin, Bin Yang, Xingyi Jin, Hang Zhao, Naijie Liu

**Affiliations:** Neurosurgery Department, China-Japan Union Hospital of Jilin University, Changchun, Jilin, China

**Keywords:** glioblastoma, cuproptosis, CP-score, prognostic model, immune response

## Abstract

Glioblastoma, a notably aggressive brain tumor, is characterized by a brief survival period and resistance to conventional therapeutic approaches. With the recent identification of “Cuproptosis,” a copper-dependent apoptosis mechanism, this study aimed to explore its role in glioblastoma prognosis and potential therapeutic implications. A comprehensive methodology was employed, starting with the identification and analysis of 65 cuproptosis-related genes. These genes were subjected to differential expression analyses between glioblastoma tissues and normal counterparts. A novel metric, the “CP-score,” was devised to quantify the cuproptosis response in glioblastoma patients. Building on this, a prognostic model, the CP-model, was developed using Cox regression techniques, designed to operate on both bulk and single-cell data. The differential expression analysis revealed 31 genes with distinct expression patterns in glioblastoma. The CP-score was markedly elevated in glioblastoma patients, suggesting an intensified cuproptosis response. The CP-model adeptly stratified patients into distinct risk categories, unveiling intricate associations between glioblastoma prognosis, immune response pathways, and the tumor’s immunological environment. Further analyses indicated that high-risk patients, as per the CP-model, exhibited heightened expression of certain immune checkpoints, suggesting potential therapeutic targets. Additionally, the model hinted at the possibility of personalized therapeutic strategies, with certain drugs showing increased efficacy in high-risk patients. The CP-model offers a promising tool for glioblastoma prognosis and therapeutic strategy development, emphasizing the potential of Cuproptosis in cancer treatment.

## 1 Introduction

Glioblastoma is a severe and short-survival brain tumor (1). Several types of research indicate that glioblastoma would be subdivided into proneural, classical, and mesenchymal molecular subtypes (2, 3). The mesenchymal exhibits more malignant physiology than the other two varieties, with a median survival of only 11.5 months. Conventional therapies for glioblastoma remain inadequate due to treatment resistance (3).

Copper regulation is essential for cellular enzyme activity, and inappropriate copper deposition can result in cell death (4).Cuproptosis is a recently found mechanism for cell death connected with mitochondria respiration (5). Cuproptosis reflects the biological disorder of copper homeostasis and can be activated by specific regulatory factor such as FDX1, the loss of which gives cell tolerance to cuproptosis (5). According to a previous study (6), FDX1 are involved in lung cancer metabolism and prognosis. Moreover, copper levels are changed in individuals with bladder or breast cancer (7), as well as glioblastoma that the copper is more significant than normal tissue (8). Computer scanning analysis revealed that glioblastoma could absorb more cupric ion than low-grade glioma (9). Copper intratumoral increases angiogenesis, facilitates immune suppression, and influences cancer development and metastasis (7, 10, 11). Copper-associated drugs have enormous therapeutic promise for cancer (12). However, this research is still in its infancy due to the lack of cancer cell selectivity and the difficulties in identifying susceptible individuals (5, 7). Bioinformatics enables the identification of the regulatory function of cuproptosis, the prediction of therapeutic medicines, and the selection of sensitive patients from large cohorts.

Here, we established and assessed the cuproptosis score in glioblastoma patients with single-cell and bulk data. Several regression methods were leveraged to explore the prognostic cuproptosis-related genes. Consequently, we developed a cuproptosis-based model to predict glioma patients’ prognosis and clinical characteristics. The risk score revealed associations between glioblastoma prognosis, immune response pathways, and the immunological microenvironment. In addition, we analyzed functional differences, immunological landscape, immune checkpoint inhibitors (ICIs), and chemotherapy response between the identified risk subgroups. As a potential novel therapy for glioblastoma, cuproptosis-targeting medicines were projected to have promise. In conclusion, our CP-model may aid in elucidating the cuproptosis-related functions and serve as a unique therapeutic tool for glioblastoma-specific treatment.

## 2 Materials and methods

### 2.1 Human specimen collection

All human specimens were meticulously selected based on stringent criteria to ensure the integrity and relevance of our research findings. The specimens comprised glioma tissues obtained from six patients undergoing surgical resection at the China-Japan Union Hospital. Only specimens from patients with histopathologically confirmed glioblastoma were included, ensuring the study focused on relevant disease pathology. Specimens were obtained from patients who had not received any form of chemotherapy or radiotherapy prior to surgery. This criterion was established to minimize the potential influence of prior treatments on the gene expression profiles of the tumor tissues. Human samples were collected with the approval of the China-Japan Union Hospital Ethics Committee. All patients signed informed consent under the declaration of Helsinki.

### 2.2 Data acquisition

Glioma patients with the gene expression profile, clinical features, and survival information were acquired from the TCGA database. Testing sets were adopted from the CGGA database (13) and GlioVis database (Rembrandt and Gill cohorts) (14). Immunotherapy cohorts were downloaded from the GEO database (GSE35640 and GSE78220 cohorts) (15, 16) and one published study (17). The single-cell profiles of glioma were acquired from the GSE182109 accession in GEO database (18).

### 2.3 Functional analysis

Go and KEGG databases were applied to perform function analysis in the clusterProfiler package (19). P value less than 0.05 is significant.

### 2.4 Establishment of cuproptosis score

We manually collected cuproptosis-associated genes from literature (5) and GSEA database (GOBP_COPPER_ION_HOMEOSTASIS.v2022 and GOBP_RESPONSE_TO_COPPER_ION.v2022) (20). To assess the cuproptosis response in glioma, we analyzed differential cuproptosis-related genes between the glioma and normal tissues and established the cuproptosis score (CP-score) based on the differential genes using the ssGSEA (for bulk data) and Ucell (for single-cell data) algorithms.

### 2.5 Construction of cuproptosis risk model

To filter out the significant prognostic genes for GBM, we performed Cox and Lasso regression based on the differential cuproptosis-associated genes. Significant genes with (Cox regression< 0.05) were retained for further analysis. To improve the model’s robustness, we randomly divided the training set into internal training and testing sets at 50% and then tested the model in three external sets. Each potential model was evaluated using 1-, 3- and 5-year AUC. Finally, the cuproptosis scoring model (CP-model) was constructed using the mathematical methodology below:


$$riskscore=\sum_{i=1}^{n}\left(\beta i_{\times }Exp_{i}\right)$$

ere n is the number of cuproptosis-related genes, exp is the gene expression profiles, and β is the Cox regression coefficient. GBM individuals were categorized by the CP-model. The predictive ability was assessed using survival analysis.

### 2.6 Evaluation of CP-model reliability

We assessed the hazard ratios (HRs) using Cox regression for CP-model and other clinical features. HRs and P-value were shown in the forest plot. We then constructed a nomogram to display the survival time of GBM individuals in indicated times. Calibration plot demonstrated the accuracy of nomogram. Finally, we evaluated the CP-model and five existing models using C-index, KM curves, and restricted mean survival time (RMST).

### 2.7 Analysis of the tumor microenvironment

We applied CIBERSORT algorithm to assess the proportions of immune cell types (21). To assess immune response and tumor cell activity, we adopted 20 pathways from a published study (22) and calculated pathway activity using the ssGSEA with the gsva R package. ESTIMATE and TIDE algorithms were applied to evaluate tumor purity and T cell dysfunction (23, 24).

### 2.8 Chemotherapeutic prediction

The GDSC database and pRRophetic package were leveraged to evaluate the drug sensitivity for each specimen in R (25). Therein, we calculated the IC50 with 10-fold cross-validation and default setting (26).

### 2.9 Immunohistochemistry

Glioma tissue samples were prepared as 4 μm sections. The slides were deparaffinized, rehydrated in a series of gradient ethanol, and recovered them by heating the tissue at 100°C in citrate buffer for 1 hour. The endogenous peroxidase activity was inhibited with 3% hydrogen peroxide for 10 minutes at room temperature. The slides were treated with primary antibodies overnight at 4°C. After washing with PBS, we then incubated it with goat serum at room temperature for 30 min. Each segment was rinsed with PBS and then incubated with DAB for 5 min. Two pathologists assessed the IHC staining. IHC was performed using antibodies against CD3 (ab16669, Abcam), FOXP3 (ab20034, Abcam), Tryptase (ab2378, Abcam), and CD163 (ab79056, Abcam).

### 2.10 Patients stratification and qPCR assay

The classification of glioblastoma patients into high-risk and low-risk groups was based on their CP-model score, which was computed from the expression levels of CP-model genes measured by quantitative PCR (qPCR). This approach allowed for a robust stratification of patients. qPCR assays were conducted to measure the expression levels of the genes comprising our CP-model. RNA was extracted from glioblastoma tissue samples using TRIzol reagent, and cDNA synthesis was carried out using a High-Capacity cDNA Reverse Transcription Kit. qPCR was performed using SYBR Green PCR Master Mix in a Real-Time PCR Detection System. The CP-model score for each patient was calculated based on the expression levels obtained from the qPCR results using the formula derived from our model. The ΔΔCt method was used to analyze the relative changes in gene expression normalized to an endogenous reference gene and relative to a control.

## 3 Results

### 3.1 Exploration of cuproptosis in glioblastoma

**Figure 1:**
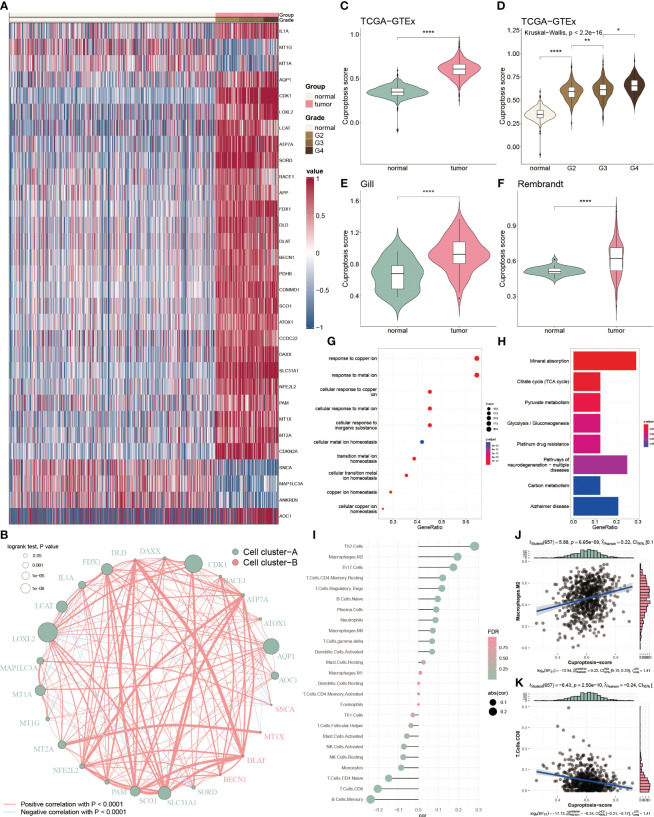
Functional enrichment and prognostic characters of cuproptosis-related genes. **(A)** Heatmap showing the 36 differential cuproptosis-related genes in glioma. **(B)** Interactive correlation of 25 cuproptosis-related genes with significant prognosis. **(C)** Distribution of CP-score in TCGA-GTEx datasets between the tumor and normal tissues. **(D)** Distribution of CP-score in TCGA-GTEx datasets with grade information. **(E, F)** Distribution of CP-score in Gill and Rembrandt datasets. **(G)** GO enrichment of differential cuproptosis-related genes. **(H)** KEGG analysis of differential cuproptosis-related genes. **(I)** Correlation of CP-score with immune cell inflictions. **(J)** Representative positive correlation: M2 macrophage. **(K)** Representative negative correlation: CD8+ T cells. *P<0.05, **P<0.01, ****P<0.0001.

To inspect the cuproptosis dysfunction in glioblastoma, we collected 65 cuproptosis-related genes from the published study and GSEA database and performed the differential analysis between glioblastoma and normal tissue. We found 31 differential expressed cuproptosis-related genes, including 26 up-regulated and 5 down-regulated genes (**Figure 1A**). To comprehensively analysis the correlation among these differential genes, we categorized them into two clusters based on hierarchical clustering and constructed a regulatory network. We observed a significant positive association of DLAT with DLD (cor = 0.703), and a negative association of MAP1LC3A with SORD (cor = -0.369) within the cluster A (**Figure 1B**).

We further constructed the CP-score to evaluate cuproptosis response in GBM. The CP-score was remarkably higher in the GBM patients versus the normal individuals (**Figure 1C**), and significantly associated with the advanced grade (**Figure 1D**). Rembrandt and Gill datasets were used to validate the results (**Figures 1E, F**). Furthermore, we performed enrichment analysis of the differential expressed cuproptosis-related genes and observed that they were enriched in multiple functions and pathways, such as copper regulation and homeostasis, metal transition pathways and mineral absorption (**Figures 1G, H**).

As tumor microenvironment (TME) are involved in tumorigenesis, we then assessed the CP-score with infiltrated immune cells in GBM. We found that CP-score were significantly correlated with several cell infiltrations (**Figure 1I**), such as positive correlation with M2 macrophages (**Figure 1J**), negative correlation with CD8^+^ T cells (**Figure 1K**).

### 3.2 Assessment of CP-score at single-cell level

**Figure 2:**
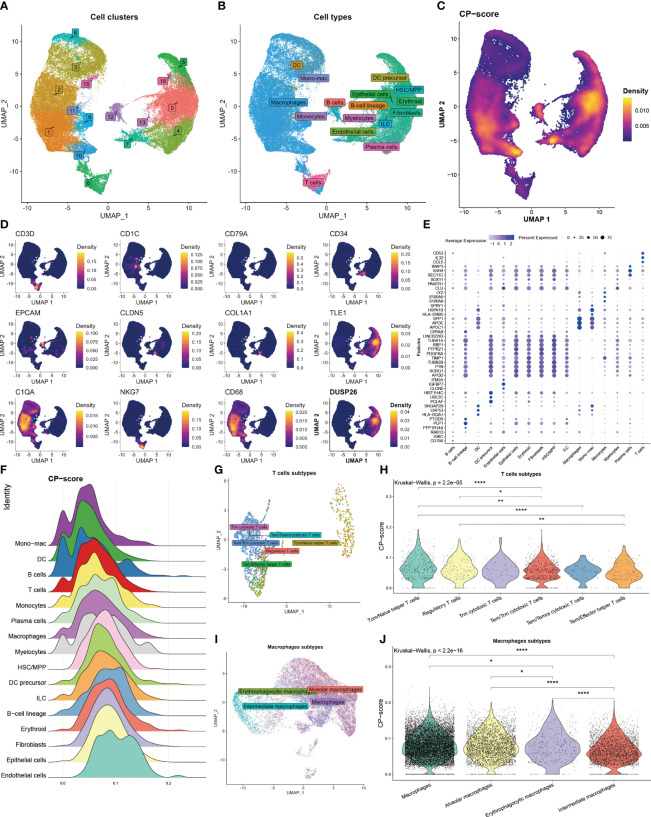
Landscape of CP-score in single-cell level. **(A)** Distribution of cell cluster. **(B)** Distribution of cell annotation. **(C)** CP-score distribution. **(D)** Representative cell marker in each cell type. **(E)** Top differential genes in each cell type. **(F)** CP-score difference among distinct cell types. **(G)** Distribution of cell annotation in T cells subtypes. **(H)** CP-score correlates with macrophage subtypes. **(I)** Distribution of cell annotation in T cell subtypes. **(J)** CP-score correlates with macrophage subtypes. *P<0.05, **P<0.01, ****P<0.0001.

To further assess the correlation between CP-score and TME in single-cell level, we acquired 60094 cells from GEO database under the GSE182109 accession number after quality control. We categorized these cells into 17 clusters (**Figure 2A**), and then annotated the cell types using the celltypist algorithm (**Figure 2B**). Moreover, the CP-score were calculated using the Ucell algorithm (**Figure 2C**). To distinguish each cell type, we labeled the representative markers of each cell type and demonstrated the top differential genes (**Figures 2D, E**). We observed that CP-score were significantly correlated with immune cell infiltration in GBM samples (**Figure 2F**).

Due to the importance of T cell and macrophages in the TME, we evaluated the CP-score in distinct T cell and macrophage subsets. We annotated the subtypes and observed the strong correlation among the cell diversity of CP-score which were in accordance with the bulk data (**Figures 2G–J**).

### 3.3 Construction of cuproptosis risk model

**Figure 3:**
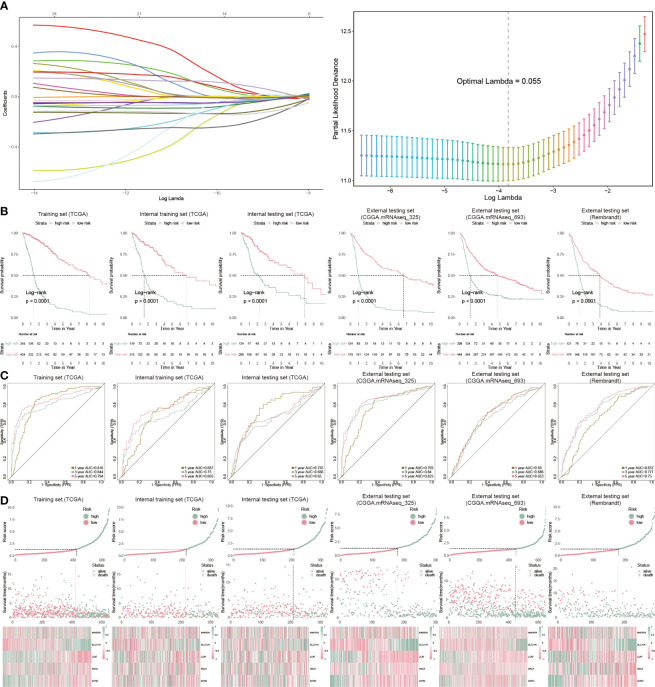
Prognostic analysis of CP-model. **(A)** Lasso regression was used to screen out significant genes. **(B)** KM survival curve in the training sets and the testing sets. **(C)** Time-dependent ROC curve analysis of the CP-score in the training sets and the testing sets. **(D)** Risk plot distribution, survival status, and relative expression of risk factors in the training sets and the testing sets.


We further analyzed cuproptosis-related genes with glioblastoma prognosis using Cox regression. Twenty-five genes were associated with glioblastoma prognosis and ten-fold cross-validation was leveraged to calculate the deviance (**Figure 3A**). To identify the genes with the highest predictive value, we performed the Cox regression with LASSO penalty in the TCGA and internal training sets using 50% random sampling. We finally got five cuproptosis genes to establish the cuproptosis risk model (CP-model) as shown below:

riskscore=ANKRD9×0.271+SLC31A1×0.724−LCAT×0.379−SNCA×0.298−SORD×0.606

We categorized GBM patients into two risk subgroups using the CP-model for the training and external sets using the same cut point obtained from the training set. As expected, we observed a shorter OS in the high-risk subtype from the TCGA training cohort as well as other five cohorts (**Figure 3B**). In addition, the predictive ability of CP-model was also evaluated using the time-dependent ROC analysis in the 1-, 3- and 5-year were all above 0.5, indicating the excellent predictive ability of CP-model (**Figure 3C**). Finally, the relative gene expression profile and risk score distribution were illustrated in **Figure 3D**. To monitor the robustness of the CP-model, the reliability of CP-model using the TCGA set was evaluated in three external sets using the same formula and cutoff from training cohort.

### 3.4 Performance of CP-model

**Figure 4:**
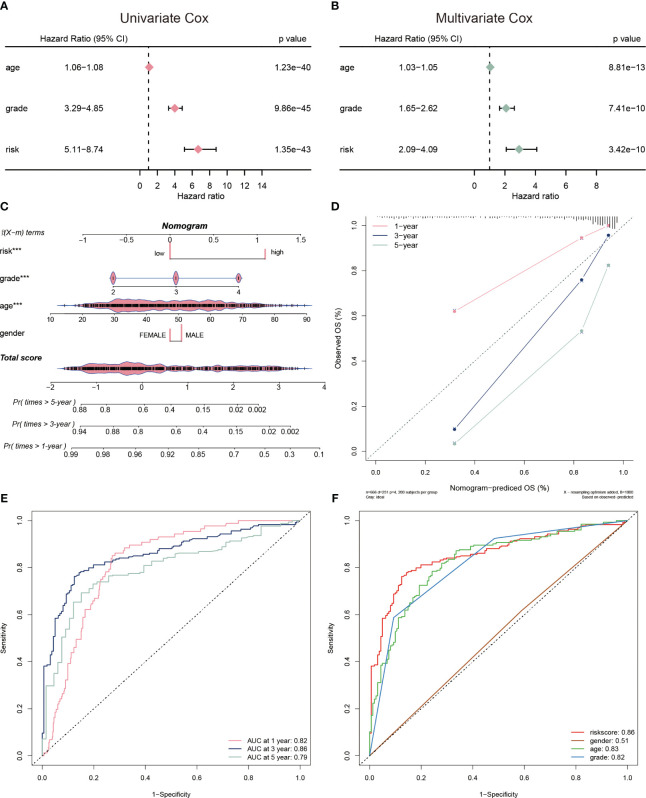
Evaluation of risk model. **(A)** The univariate Cox regression analyses of the risk score and other clinical factors. **(B)** The multivariate Cox regression analyses of the risk score and other clinical factors. **(C)** Nomogram was used to predict 1-, 3-, and 5-year OS of glioblastoma. **(D)** Calibration curves was used to demonstrate the nomogram−predicted OS and observed OS of glioblastoma patients. **(E)** ROC curve demonstrating the AUC at 1-, 3-, and 5-year for the risk score. **(F)** ROC curve demonstrating the AUC of risk score and other clinical factors.

To evaluate the clinical application of the CP-model, we performed univariate and multivariate analyses with some clinical vicariates. We observed that the CP-model demonstrated a more reliable for glioblastoma prognosis compared with age and grade (**Figures 4A, B**). In addition, a nomogram was built to help clinicians to analyze the survival probability of GBM individuals by combining the CP-model and other independent factors (**Figure 4C**). In brief, these factors were weighted using a point system. Assigning values to the variables included drawing an upward-sloping straight line and then rescaling the total of the points for each variable. The total points were calculated by adding together all the individual variable scores. Illustrating a straight line to the outcome axis allowed us to calculate the 1-, 3-, and 5-year survival probabilities for GBM. The calibration plot revealed that the bias-corrected line was near the ideal curve, indicating high degrees of concordance between the predictive and actual points (**Figure 4D**). We further applied a time-dependent ROC curve to assess the survival probability, the AUC were 0.82, 0.86, and 0.79 in 1-, 3-, and 5-year, respectively (**Figure 4E**). The risk scores were a better predictor of outcomes than traditional factors (**Figure 4F**).

**Figure 5:**
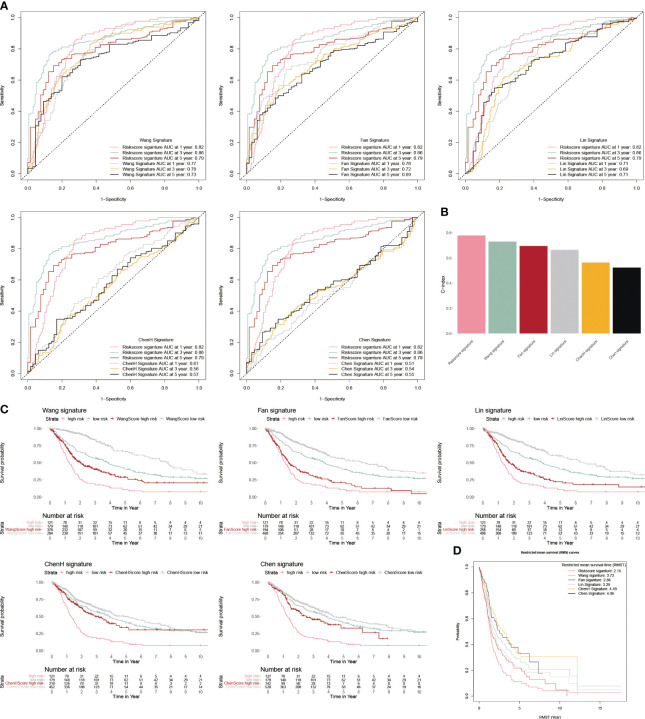
Comparison of CP-model. **(A)** The ROC curve of Wang, Fan, Lin, ChenH, and Chen signatures. **(B)** Barplot demonstrating the C-index of six risk signatures. **(C)** The KM survival curve of Wang, Fan, Lin, ChenH, and Chen signatures. **(D)** RMST for each of the six models.

We chose five popular risk models from the literature to evaluate against our CP-model (27–31), and we plotted their ROC and KM curves to show how they compare (**Figures 5A, C**). C-index was calculated using the rms package in R. According to the findings, the C-index for CP-model was higher than other five predictive risk models (**Figure 5B**). The high-risk subgroup’s RMST across all six models is shown in **Figure 5D**. Overall, our results indicate that the CP-model was more accurate for predicting glioblastoma patients’ survival than individual prognostic markers and other existing models.

### 3.5 Clinicopathological significance of the CP-model

**Figure 6:**
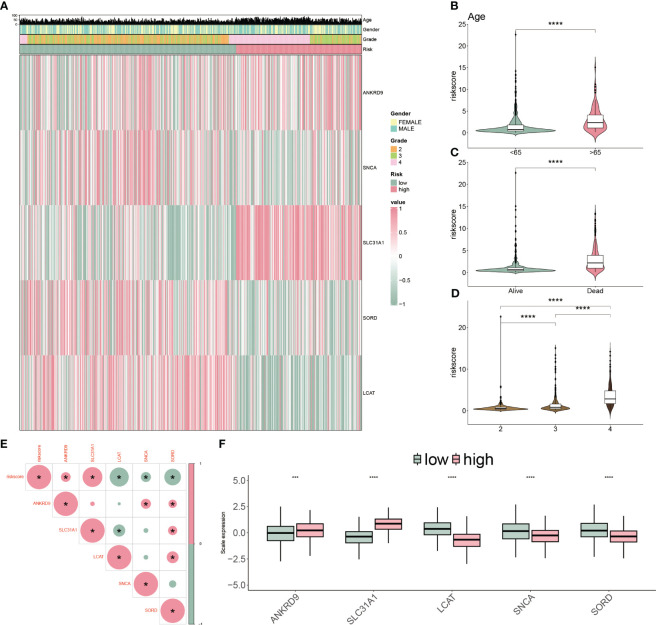
Clinicopathological characteristics of CP-model. **(A)** Heatmap demonstrating the distribution of clinical factors (age, gender and stage) and relative expression of five cuproptosis regulators in the two risk subgroups. **(B)** The scatter diagram of risk score and age. **(C)** The scatter diagram of risk score and survival status. **(D)** The scatter diagram of risk score and grade. **(E)** The association of five cuproptosis regulators with the risk score. **(F)** Relative expression of five cuproptosis regulators between the two risk subgroups. ***P< 0.01, ***P< 0.001.

We applied a heatmap to show the expressed profiles of five cuproptosis-related genes and clinicopathologic factors (**Figure 6A**). The expression of SNCA, SORD and LCAT were inhibited, and ANKRD9 and SLC31A were relatively higher in the higher risk patients. Furthermore, most of the patients in grade III/IV were categorized as high-risk subgroups, reflecting the feasibly predictive ability of the CP-model. Risk scores were also associated with age, dead status, and grade in glioblastoma (**Figures 6B–D**). In line with risk stratification, the risk score was correlated positively with ANKRD9 and SLC31A, but negatively with SNCA, SORD and LCAT (**Figures 6E, F**). Altogether, our results indicated that the CP-model was independent of conventional clinical characteristics.

### 3.6 Functional variations between the CP-model subtypes

**Figure 7:**
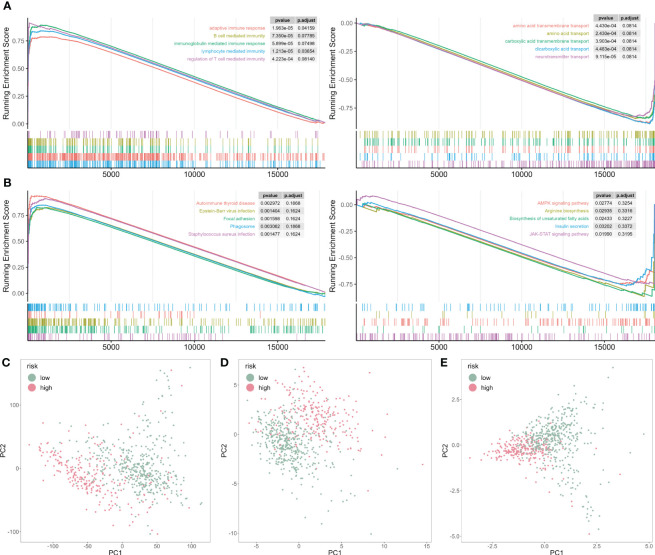
GSEA analysis of risk score-associated functions. **(A)** Representative GO enrichment between the two risk subgroups. **(B)** Representative KEGG enrichment between the two risk subgroups. **(C)** PCA for all gene expression profile. **(D)** PCA for all cuproptosis-related genes expression profile. **(E)** PCA for five cuproptosis-related genes expression profile.

To mechanically detect the difference between the two subtypes, we applied GSEA analysis based on GO and KEGG databases. We observed that some GO functions were activated in the high-risk patients, such as adaptive immune response, B cell and T cell-mediated activation (**Figure 7A**). However, some GO functions were inhibited, including multiple amino acid transport functions (**Figure 7A**). In addition, multiple disease-related pathways were activated for KEGG analysis, but AMPK and JAK-STAT signaling pathways were inhibited (**Figure 7B**).

Next, we analyzed the principal component analysis (PCA) using the full set of genes (**Figure 7C**), on all cuproptosis genes (**Figure 7D**), and five selected cuproptosis genes from the CP-model (**Figure 7E**). We showed that the two risk subgroups were distinguishable based on the expression patterns of the selected cuproptosis regulatory genes.

### 3.7 CP-model correlates immune landscape

**Figure 8:**
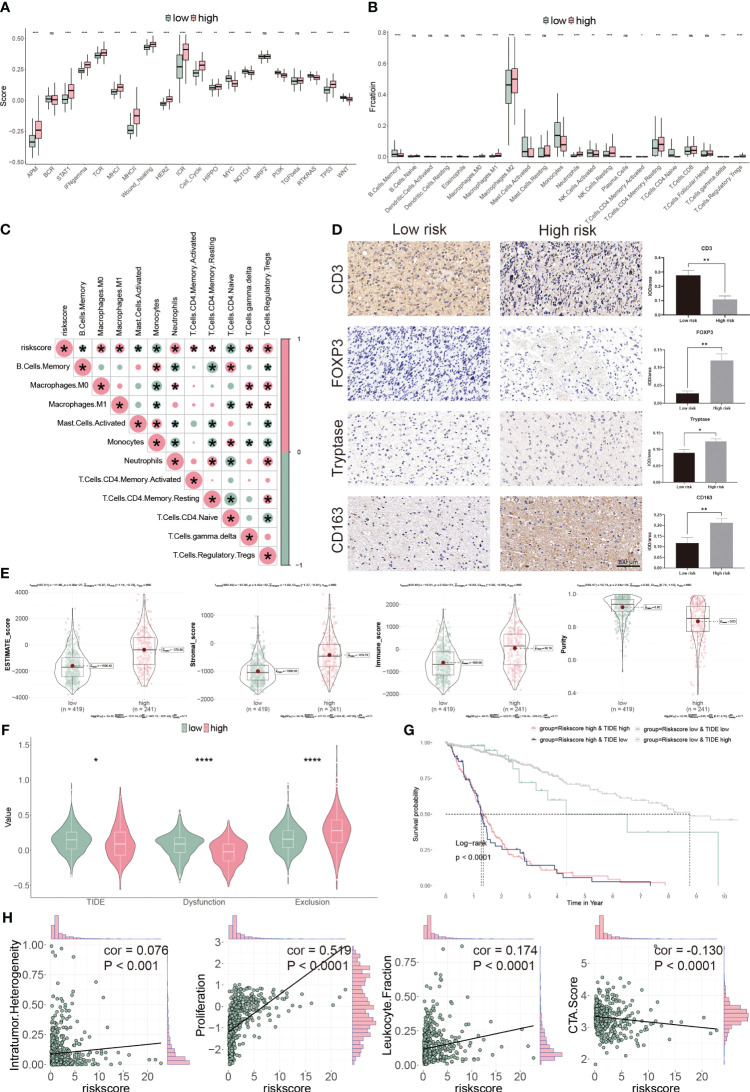
Immune landscape of CP-model. **(A)** Pathway activities between the two risk subgroups. **(B)** Differential immune infiltration of 22 immune cell fractions between the two risk subgroups. **(C)** The correlation of 22 immune cell types with the risk score. **(D)** Representative IHC images of immune cell markers between the two risk subgroups. **(E)** Correlation of risk score with tumor microenvironment. **(F)** TIDE, T cell dysfunction and exclusion between the two risk subgroups. **(G)** Survival analysis of patients with different combinations of risk scores and TIDE in TCGA cohort. **(H)** Correlation of risk score with the intratumor heterogeneity, cell proliferation, leukocyte fraction and CTA score. *P< 0.05, **P< 0.01, ***P< 0.001, ****P<0.0001, n.s, not significant.

To assess the TME in glioblastoma patients, we calculated immune response and tumor cell activity using the ssGSEA and assessed the infiltrated immune cell using the CIBERSORT algorithm. We found that 17 pathways and 14 types of cells were dramatically altered between the two subgroups (**Figures 8A, B**). Moreover, the risk score was positively correlated with seven cells, but negatively correlated with four cells (**Figure 8C**). Finally, we validated our findings using IHC by targeting immune cell markers (**Figure 8D**). We confirmed that CD3 targeting T cells were accumulated in low-risk group patients. However, FOXP3 targeting Tregs, tryptase targeting mast cells and CD163 targeting M2 macrophage were collected in high-risk group patients.

We next evaluated the tumor heterogeneity using ESTIMATE. High-risk subgroup patients have significantly elevated estimate, stromal, and immune scores but have low tumor purity (**Figure 8E**). Moreover, TIDE and T cell dysfunction, but not exclusion, were substantially lower in the high-risk subgroup (**Figure 8F**). Patients with low-risk and high TIDE scores exhibited the more extended OS than the others (**Figure 8G**). High-risk score correlated with high intratumor heterogeneity, high proliferation activity, high leukocyte fraction and low CTA score (**Figure 8H**). Thus, our findings show that aberrant immunological infiltration and tumor heterogeneity in glioblastoma may serve as prognostic markers and immunotherapy targets and may have substantial therapeutic consequences.

### 3.8 CP-model predicts immunotherapy and chemotherapy in GBM

**Figure 9:**
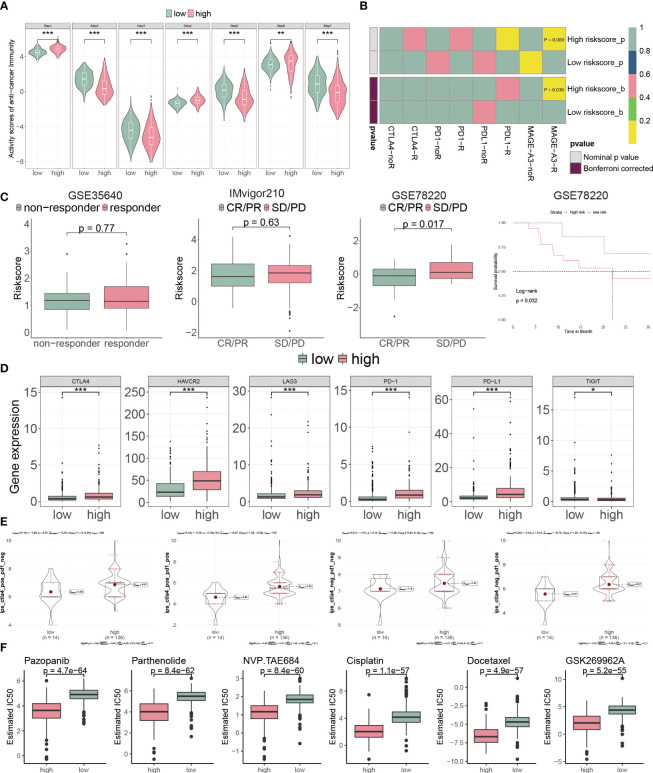
CP-model predicted the response to chemotherapy and targeted therapy. **(A)** Differential immune cycle processes between the two risk subgroups. **(B)** Putative immunotherapy response between the two risk subgroups. **(C)** Risk score distribution for different immunotherapy target responses in the GSE35640 cohort, IMvigor210 cohort and GSE78220 cohort. **(D)** Differential expression of six immunosuppressive molecules between the two risk subgroups. **(E)** Four subtypes of IPS values between the two risk subgroups. **(F)** Drug sensitivity of Pazopanib, Parthenolide, NVP.TAE684, Cisplatin, Docetaxel and GSK269962A between the two risk subgroups. *P< 0.05, **P< 0.01, ***P< 0.001, n.s, not significant.

Cancer immunity cycle is a set of sequential events that killing cancer cells (32). The high-risk subgroup patients had a low score in steps 2 3 and 5, compared with the low-risk ones (**Figure 9A**). Conversely, the high-risk group presented low score in steps 1, 4, and 6 (**Figure 9A**). These findings indicated that high-risk patients had a better ability to identify tumors and induced T cells to tumors, but a weaker ability to infiltrate immune cells and kill tumors (32).

Next, we assessed the immunotherapeutic prediction of the CP-model using the SubMAP algorithm. High-risk score patients would get a better effect from anti-MAGA-A3 treatment (**Figure 9B**). However, we do not observe significant responsiveness in anti-PD-1/PD-L1/CTLA4 treatments. We further detailed the immunotherapeutic responsiveness in a different cohort from GSE35640 (targeting MAGE-A3 immunotherapy), IMvigor210 (targeting PD-L1 immunotherapy) and GSE78220 (targeting PD-1 immunotherapy). Consistent with the SubMAP results, the risk score could be negatively associated with the anti-MAGA-A3 response. Higher score patients presented worse outcomes in the GSE35640 cohort (**Figure 9C**). In addition, the risk score was positively associated with ICIs, such as PD-1, PD-L1, CTLA4, HAVCR2, LAG3 and TIGHT (**Figure 9D**). Interestingly, the CP-model could predict the response to the combination treatment of anti-PD-1 and anti-CTLA4 (**Figure 9E**). Since chemotherapy is a common way of anticancer, we analyzed the CP-model with chemotherapy response. We found that Pazopanib, Parthenolide, NVP.TAE684, Cisplatin, Docetaxel and GSK269962A would be more effective in high-risk patients (**Figure 9F**).

## 4 Discussion

Although glioblastoma is the common cancer of brain, its prognosis differs amongst molecular subgroups. There is an urgent need for innovative and efficient methods to assess and improve the prognosis of glioblastoma. Our understanding of how cancers cause cell death is constantly refined as new programmed cell death patterns are discovered and associated molecular pathways are elucidated. Tsvetkov et al. recently created the term “cuproptosis” to describe a new kind of copper-dependent apoptosis (5). Several scholarly articles have established prognostic models based on cuproptosis-related genes. These models highlight the potential of leveraging copper metabolism pathways as biomarkers for glioblastoma prognosis and therapeutic targets (33–36). However, the precise mechanism of cuproptosis-related genes is currently unknown. In the present study, five cuproptosis genes were evaluated for associations with cuproptosis, and the results were used to develop a risk signature successfully. These genes were ANKRD9, SLC31A1, LCAT, SNCA and SORD. Additionally, we demonstrated that patients belonging to high-risk groupings based on target cuproptosis-related genes are significantly associated with shorter OS, a weaker immunological effect, and increased chemosensitivity compared to the low-risk cohort.

Immunotherapy has proven an effective cancer treatment method in clinical practice (37). Immunotherapy that blocks the PD-1/PD-L1 checkpoint has joined chemotherapy as the conventional medicine for lung cancer (38). Unfortunately, the metabolic reprogramming of tumors presents a formidable obstacle for immune cells to fulfill their jobs and for cancer immunotherapy to be effective (39). Specifically, cells with a high concentration of lipid-acylated proteins are more susceptible to copper-induced cell death. Thus, in cancers characterized by an increased lipid metabolism, the stimulation of copper-mediated cell death may eliminate medication resistance from immune evasion. As anticipated, our GO and KEGG analyses revealed strong associations between immune response-related pathways. Our CP-model indicated an inextricable link between copper-dependent cell death and tumor immune responses in glioblastoma when seen as a whole (40). Moreover, we have extended our analysis to include recent findings by Zhu et al., which highlight the prognostic significance of cuproptosis clusters in low-grade glioma (LGG) and their correlation with immunotherapy response (41). This study’s approach to categorizing patients based on cuproptosis-related DEGs mirrors our methodological framework in glioblastoma, underscoring the potential universality of cuproptosis as a prognostic marker across glioma subtypes. The evidence suggesting that cuproptosis-related genes influence patient prognosis by modulating immune cell infiltration and tumor-associated pathways further supports our hypothesis that targeting these pathways could offer new therapeutic avenues. Inspired by these findings, we posit that our CP-model not only serves as a reliable predictor for glioblastoma patient outcomes but also holds the potential to guide immunotherapeutic strategies, enhancing the precision of treatment modalities in glioblastoma.

Since glioblastoma typically does not react favorably to ICIs, it is considered immunologically “cold” (42). The high percentage of myeloid cells (30-50%) within glioblastoma tumors that act to inhibit the immune cells entering the tumor is primarily responsible for this immune escape (43). One of the primary components of the glioblastoma TME is myeloid-derived suppressor cells (MDSCs), which limit cytotoxic T lymphocyte and natural killer cell activity (43). In addition, CTLA4 (44), PD-1 (45), and TIM3 (46) are intensively researched checkpoint molecules in glioblastoma. Given that PD-L1 expression levels are inversely associated with clinical outcome (47), we explored the expression of important role in the immune checkpoints, such as PD-1/PD-L1, CTLA4, HAVCR2, LAG3 and TIGHT, to better understand the connections between the CP-model and immune invasion. Our data demonstrated a strong correlation between the risk score and the expression of immunological checkpoints, with higher expression levels observed in the high-risk subgroup, which also had a worse outcome. These findings suggest that elevated immune checkpoint expression contributes to a more immunosuppressive milieu, which promotes glioblastoma progression in the high-risk subgroup and may explain why this group has a worse prognosis.

Our study introduces a novel prognostic model for glioblastoma, advancing the understanding of cuproptosis beyond the scope of existing models. Furthermore, our paper builds upon the groundwork laid by previous studies by offering innovative approaches and insights. Importantly, we have developed a prognostic model with refined predictive accuracy for glioblastoma outcomes by integrating a unique set of cuproptosis-related genes. Additionally, our model is thoroughly validated using both single-cell and bulk RNA-seq data, providing a multi-dimensional perspective that reinforces the model’s applicability and robustness. In particular, we delve deeper into the interplay between cuproptosis-related genes and the immune microenvironment, a connection that has not been extensively explored in glioblastoma. Consequently, our CP-model correlates with both the immunological landscape and chemotherapy responses, paving the way for its use as a clinical tool for personalized treatment strategies. Lastly, we employ advanced computational techniques, including machine learning algorithms, to develop a prognostic score that outperforms traditional clinical parameters and previous prognostic models.

## 5 Conclusion

Our CP-model was shown to be an independent predictive biomarker for patients with glioblastoma by examining the immune landscape of glioblastoma from multiple cohorts. Genes enriched in the immune response and immunological-related pathways were most prominent among those revealing variations in immune infiltration across subgroups. In addition, a nomogram was built that quantitatively predicts glioblastoma patients’ OS by merging the CP-model and clinical parameters. The CP-model foretells the effectiveness of immunotherapy and chemotherapy in treating glioblastoma. Therefore, it aids doctors in making judgments about patient care.

## Data availability statement

Publicly available datasets were analyzed in this study. This data can be found here: The data supporting this study’s findings are available from the corresponding author upon reasonable request.

## Ethics statement

The studies involving humans were approved by the Ethics Committee of China-Japan Union Hospital of Jilin University. The studies were conducted in accordance with the local legislation and institutional requirements. The participants provided their written informed consent to participate in this study.

## Author contributions

ZQ: Writing – original draft, Visualization, Resources, Methodology, Formal analysis, Data curation, Conceptualization. BY: Writing – original draft, Methodology, Formal analysis, Data curation. XJ: Writing – original draft, Visualization, Resources, Methodology, Investigation. HZ: Writing – original draft, Validation, Methodology, Investigation, Data curation. NL: Writing – review & editing, Writing – original draft, Visualization, Resources, Funding acquisition, Conceptualization.
